# The agar diffusion scratch assay - A novel method to assess the bioactive and cytotoxic potential of new materials and compounds

**DOI:** 10.1038/srep20854

**Published:** 2016-02-10

**Authors:** Mascha Pusnik, Minire Imeri, Grégoire Deppierraz, Arie Bruinink, Manfred Zinn

**Affiliations:** 1 Institute of Life Technologies, HES-SO Valais-Wallis, CH-1950 Sion, Switzerland; 2 Laboratory for Biointerfaces, Swiss Federal Laboratories for Materials Science and Technology - Empa, CH-9014 St. Gallen, Switzerland

## Abstract

A profound *in vitro* evaluation not only of the cytotoxic but also of bioactive potential of a given compound or material is crucial for predicting potential effects in the *in vivo* situation. However, most of the current methods have weaknesses in either the quantitative or qualitative assessment of cytotoxicity and/or bioactivity of the test compound. Here we describe a novel assay combining the ISO 10993-5 agar diffusion test and the scratch also termed wound healing assay. In contrast to these original tests this assay is able to detect and distinguish between cytotoxic, cell migration modifying and cytotoxic plus cell migration modifying compounds, and this at higher sensitivity and in a quantitative way.

Biocompatibility describes the capacity of a material to accomplish its intended function without inducing a negative response in a host organism, the negative response either being a local or systemic effect^1^. Thus, adequate methods are required during the R&D phase of a novel material or compound to ensure its safety and tolerance to the body. Consequently, evaluating biocompatibility should not only involve the assessment of cytotoxicity but also bioactivity of a given test material^2^. Since many years, the ISO 10993-5 guidelines represent the standard for such *in vitro* biocompatibility determination^3^. The agar overlay test, a method included in this guideline, allows to measure cytotoxicity by indirect contact^4^. In this assay a sub-confluent cell culture, e.g., mouse fibroblasts, is overlaid by agar. Thereafter, the test material is placed centrally on top of the thin layer of agar, covering around 10% of the surface of the cell culture. As a result, cells are exposed to a concentration gradient of the released compound due to the radial diffusion in the agar with highest concentration and by the strongest effects below the test sample. The agar overlay test allows only a qualitative assessment of cytotoxicity; the degree of reactivity of a given test material is solely evaluated by grading the size of the zone of dead cells around the sample by means of cell morphology and/or by a selective staining of living and/or dead cells. The limits of the current test are found in the fact that it is purely qualitative and that only acute cytotoxic effects of released compounds can be detected. Released compounds which affect cell functionality without being cytotoxic will not be identified with this assay.

The ability of certain cells to migrate is essential in many physiological processes, such as tissue repair and regeneration, as well as immune system responses^5^. *In vitro*, cell migration can be affected by numerous different alterations in cell physiology, e.g., gene expression^6^,^7^,^8^,^9^,^10^, signalling^11^,^12^,^13^,^14^,^15^, and/or a modified interaction with the extracellular matrix^16^,^17^,^18^. Therefore, using cell migration as a bioactivity indicator allows evaluating cell performance in a quantitative, qualitative, and time-dependent manner. Today cell migration assays are mainly applied in the field of wound healing, angiogenesis and anti-cancer drug research to assess bioactivity of compounds promoting migration but also to investigate toxicity or migration inhibition, respectively. Many different tests are described to evaluate the migratory potential of a compound (reviewed in^19^,^20^). One of the most prominent is the scratch assay, also known as the wound healing assay^21^. Shortly, a gap, called scratch or artificial wound, is generated by scraping and as a result removing a lane of cells within a confluent cell monolayer. Cells on the edge of this gap will migrate into the cell-free space until cell-cell contacts limit further migration. Depending on the compound concentration in the culture medium, the rate of migration might differ, which is evaluated by comparing microscopic pictures. Beside the advantage of being simple, it has some clear drawbacks. One key limitation is its inability to establish a chemical gradient of the test compound within the cell culture dish. Thus, to determine concentration-effect relationships many cultures have to be evaluated; each exposed to a different test compound concentration or material extract dilution. Another key limitation is certainly the disperse migration pattern of the cells which makes it rather difficult to perform an accurate quantitative analysis of the migrated distance. The latter is only feasible by single-cell tracking using time-lapse microscopy and the appropriate image analysis software^22^,^23^.

In this work we present a novel assay termed agar diffusion scratch assay, which combines the advantages of the agar diffusion test and the scratch assay, and additionally rules out both above mentioned key limitations. In addition, it remains a simple, low-cost assay and may be used for a broad range of applications like biocompatibility and cytotoxicity testing as well for more specific fields like cancer and angiogenesis research.

## Results

### Strategy of the agar diffusion scratch assay

**Figure 1:**
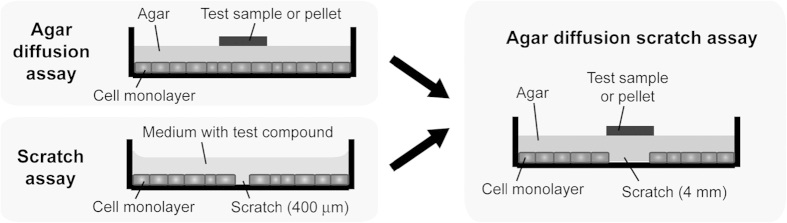
Schematic representation of the agar diffusion scratch assay in relation to the ISO 10993-5 agar diffusion assay and the scratch assay. Unlike the agar overlay test a lane without cells (scratch) and unlike in the scratch assay an agar overlay instead of culture medium is present. Cell migration takes place below the thin layer of agar and allows the testing of drug loaded pellets as well as solid test samples.

To provide a convenient and sensitive system for the quantitative evaluation of cell functionality including cell death, a novel assay was developed by combining the ISO10993-5 agar diffusion and the scratch test (Fig. 1). For this, a confluent monolayer of cells is cultured in 6-well plates. After generation of a wide, cell free, and diametrically located lane within this culture, the cell culture is overlaid by a thin layer of agar covering the cells and the gap. As a model for a test material a circular agar gel pellet of 10 mm diameter and around 5 mm thickness is prepared by mixing agar solution with the test compound or material extract prior moulding. This pellet is then placed centrally on top of the agar overlay covering about 10% of the surface in analogy to the agar diffusion test and thus releasing the cytotoxic and/or bioactive compound. The resulting concentration gradient of the test compound will eventually reach cytotoxic levels resulting in cell death and absence of cell migration. At the border of this toxic regime another regime can be distinguished in which no cytotoxic but still bioactive levels are reached. In this bioactive regime the change in cell functionality may evoke either decreased or increased cell motility. This aspect is additionally assessed in this assay. Before validating this assay the current set-up was optimized for different cell lines regarding agar layer thickness, agar concentration and duration of the test.

### Optimizing agar concentration and volume

**Figure 2:**
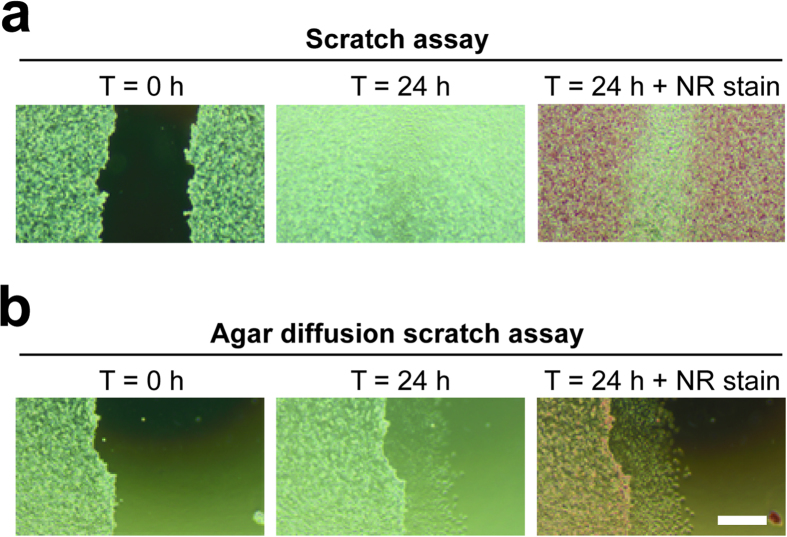
Agar diffusion scratch assay allows easy migration tracking for a prolonged period of time. Comparison of the classical scratch assay (**a**) and the novel agar diffusion scratch assay (**b**). In contrast to the scratch assay, cells were overlaid with agar in the agar diffusion scratch assay. Pictures are shown before and after 24 h of incubation. In the third column, viable cells were stained with Neutral Red (NR). The scale bar in the lower right picture represents 200 μm.

The optimal volume and concentration of the agar overlay was assessed using mouse fibroblast 3T3 and L929 cell line cells, which are the most commonly used cell lines for ISO cytotoxicity testing. After generation of a cell-free lane by scratching with a micropipette tip, cultures were either covered by 1 to 5 ml agar (1%) or just with culture medium. Migration distance was assessed from pictures of the cultures which were taken at 8, 24, 48 and 72 h after addition of the agar layer (Supplementary Fig. 1). First of all the coverage by agar positively affected the appearance of the cell migration front line. In the presence of the agar a nearly closed front line of migrating cells was seen (Fig. 2), whereas in absence of agar many single cells were rapidly migrating into the cell free gap followed by slowly migrating cells, thus enabling a more accurate measurement of the cell front line dislocation (CFD). Additionally, the generation of a wider cell free lane using a silicon scraper does not limit the evaluation of CFD to only 24 h like in the scratch assay. The concentration of the agar affected especially the 3T3 cells with high concentrations reducing CFD velocity. The CFD distance relative to t = 0 h (ΔCFD) increased throughout the 72 h of incubation (Supplementary Fig. 1). Light microscopic examination of the cells did not give evidence for toxic effects in any of the cultures taking neutral red uptake as parameter. After 48 h of incubation a clear difference in ΔCFD between the different volumes was seen with 3 ml and 4 ml agar leading to the highest ΔCFD values. Since the lowest volume (3 ml) leading to the smallest distance between test sample and cell layer this volume was seen as optimal for the agar diffusion scratch assay. Interestingly, our results suggest a difference in ΔCFD progression between the two cell lines, indicating that L929 cells migrate not only 2–3 fold faster (about 2 fold in the period between 24 and 48 h and 3 fold between 48 and 72 h after start) but also for a longer time than 3T3 cells. In contrast to L929, ΔCFD increased only negligible in case of 3T3 cells between 48 and 72 h of incubation. Next, we tested the influence of the agar concentration on cell migration, evaluating the overlay with 3 ml agar at different concentrations (0.5–2%) (Supplementary Fig. 2). While cell viability was not affected at all, cell migration tends to be decreased at low (0.5 and 0.75%) and high (>1.5%) agar concentrations. Based on evaluation and comparison of cell migration behaviour, optimal compound diffusion and besides this easy handling the 1% agar was defined as the optimal agar concentration. Furthermore, of both evaluated cell lines L929 showed maximal ΔCFD values throughout the testing period. Since alterations of ΔCFD are expected to be seen first using L929, this cell line was taken for the subsequent validations.

### Assessing cytotoxic potential of compounds

Cadmium sulphate (CdSO_4_), a commonly used positive control in cytotoxicity assays^24^, was chosen as test compound to test if the novel assay can be applied for cytotoxicity determination. For our test, agar pellets containing different concentrations of CdSO_4_ were placed on top of the agar overlay and ΔCFD’s were assessed after 24, 48, and 72 h of incubation.

**Figure 3:**
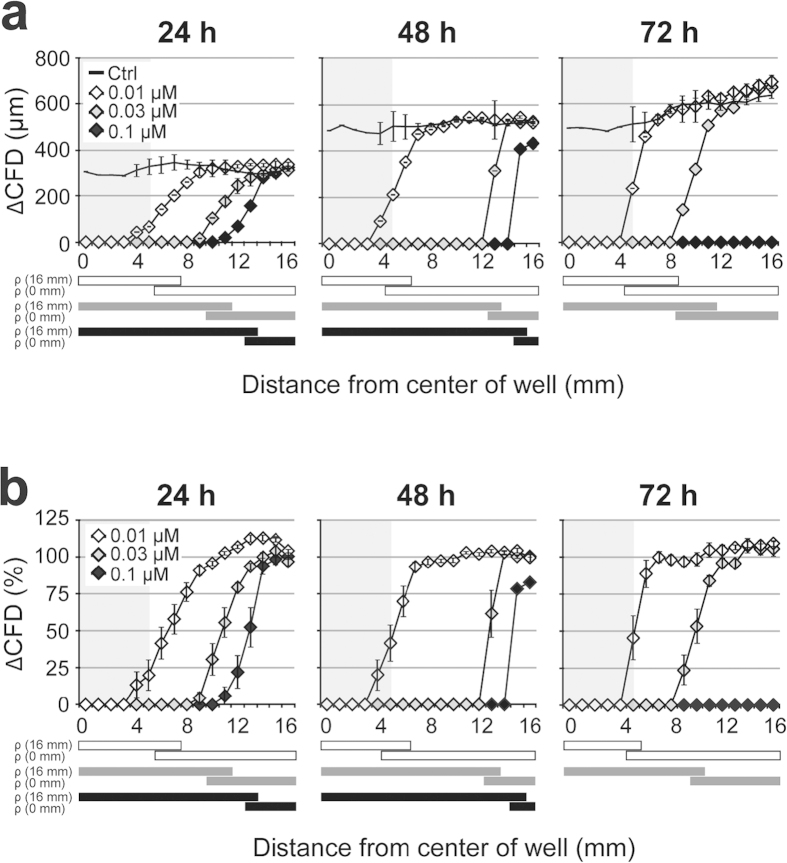
Effects on cell migration indicates cytotoxicity in a dose and time dependent manner. (**a**) Pellets with three different concentrations of CdSO_4_ were placed on top of the agar overlay. After 24, 48, and 72 h, pictures were taken at each mm along the scratch, on both sides and two migration distances were measured per picture. In addition, viability was assessed for each time point with Neutral Red. (**b**) Same as for (**a**) but here the migration distances are normalized to the respective control values. Experiments were run in triplicates and data represents mean ± S.E.M. over the mean experimental data. Significant effects (*P* < 0.05) are indicated with the bars below the graph. *P* < 0.05 compared with the migration distance at 16 mm (ρ (16 mm)) or at 0 mm (ρ (0 mm)). Significant effects for 0.01, 0.03, and 0.1 μM CdSO_4_ are indicated with white, grey and black bars, respectively.

**Figure 4:**
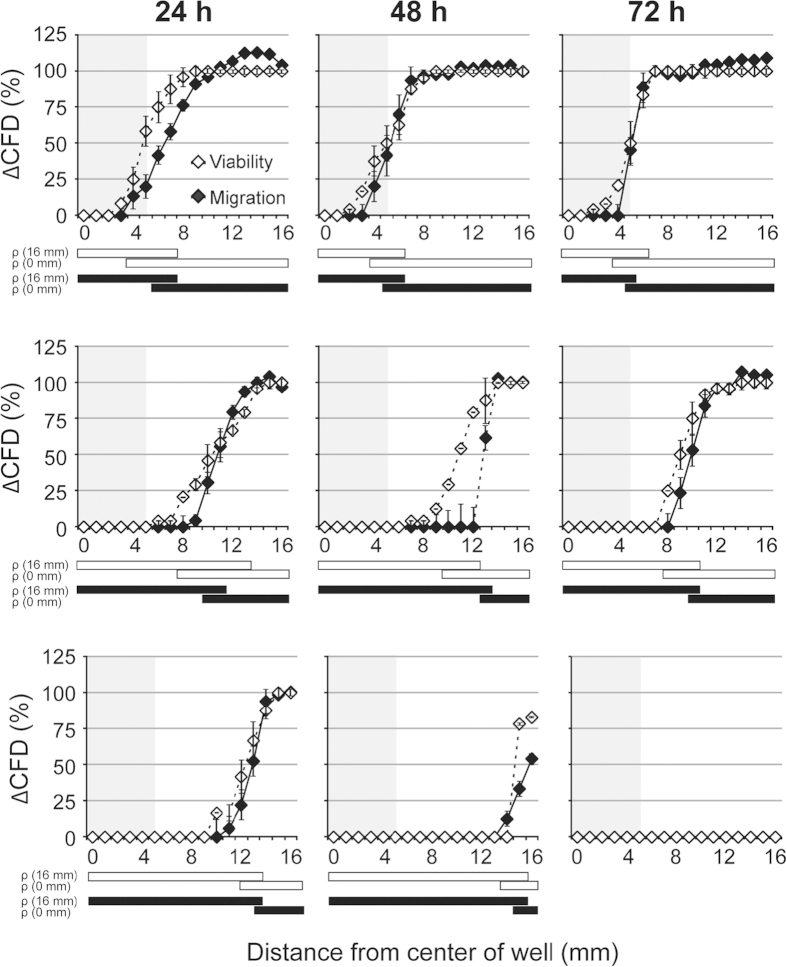
Cell migration is a sensitive indicator of bioactive compounds. Migration behavior of the cells is compared with the viability evaluation (dashed line) by NR. Migration distances were normalized to the respective control values. The viability was determined by eye, estimating the percentage of stained cells at each position of the scratch. The grey square indicates the area covered by the agar pellet. Experiments were run in triplicates and data represent mean ± S.E.M. over the mean experimental data. Bars below the graph indicate *P* < 0.05 compared with the migration distance at 16 mm (ρ (16 mm)) or at 0 mm (ρ (0 mm)). Significant effects for viability and migration results are represented with white and black bars, respectively.

As shown in Fig. 3a ΔCFD is affected by CdSO_4_ in a concentration- and time-dependent manner. With increasing CdSO_4_ concentration also the distance between projected center of the pellet and the location, where significant ΔCFD is observed (ΔC-CFD), is incremented. Strikingly, the latter distance increases with time for a given CdSO_4_ concentration, suggesting that cells that have once migrated are disintegrated or detached from the cell culture dish. Cadmium has been previously shown to induce an increase in reactive oxygen species (ROS) formation^25^, DNA damages^26^,^27^ as well as apoptosis^28^,^29^, and thus leading to collapsing cells. It has been noticed that CFD seemed to be slightly affected in the control cultures with agar pellets having no compound. One explanation could be the reduced oxygen tension at the cell surface at locations covered by the agar pellet and thus leading to less ROS. In order to correct for this effect, we normalized our values with the controls, i.e. we expressed ΔCFD values relative to the corresponding ΔCFD values of the controls determined at the same time point and distance to the projected centre of the pellet (Fig. 3b). No differences were observed between the classical qualitative agar diffusion parameters, viability of the cells taking neutral red uptake as an index, and our agar diffusion scratch assay results (Fig. 4). Strikingly, we could observe for all tested concentrations that the cytotoxicity measurement followed by ΔCFD is more sensitive, i.e. changes are seen at slightly larger distances to the projected center of the test specimen. These results suggest that CFD can not only be used as a measure of cell death but also sense adverse effects on cell functionality in general, hence implying a potential applicability of ΔCFD as a sensitive bioactivity marker. In case of CdSO_4_ the ranges, where the compound is cytotoxic and where it is bioactive were largely overlapping.

### Assessing the bioactive potential of compounds

In order to prove that the agar diffusion scratch assay is able to identify potentially bioactive compounds, we evaluated substances which are bioactive and at much higher concentrations cytotoxic. For the present study we selected salinomycin and cytochalasin D as potential negative influencers of CFD and basic fibroblast growth factor (bFGF) as potentially CFD stimulating compound.

**Figure 5:**
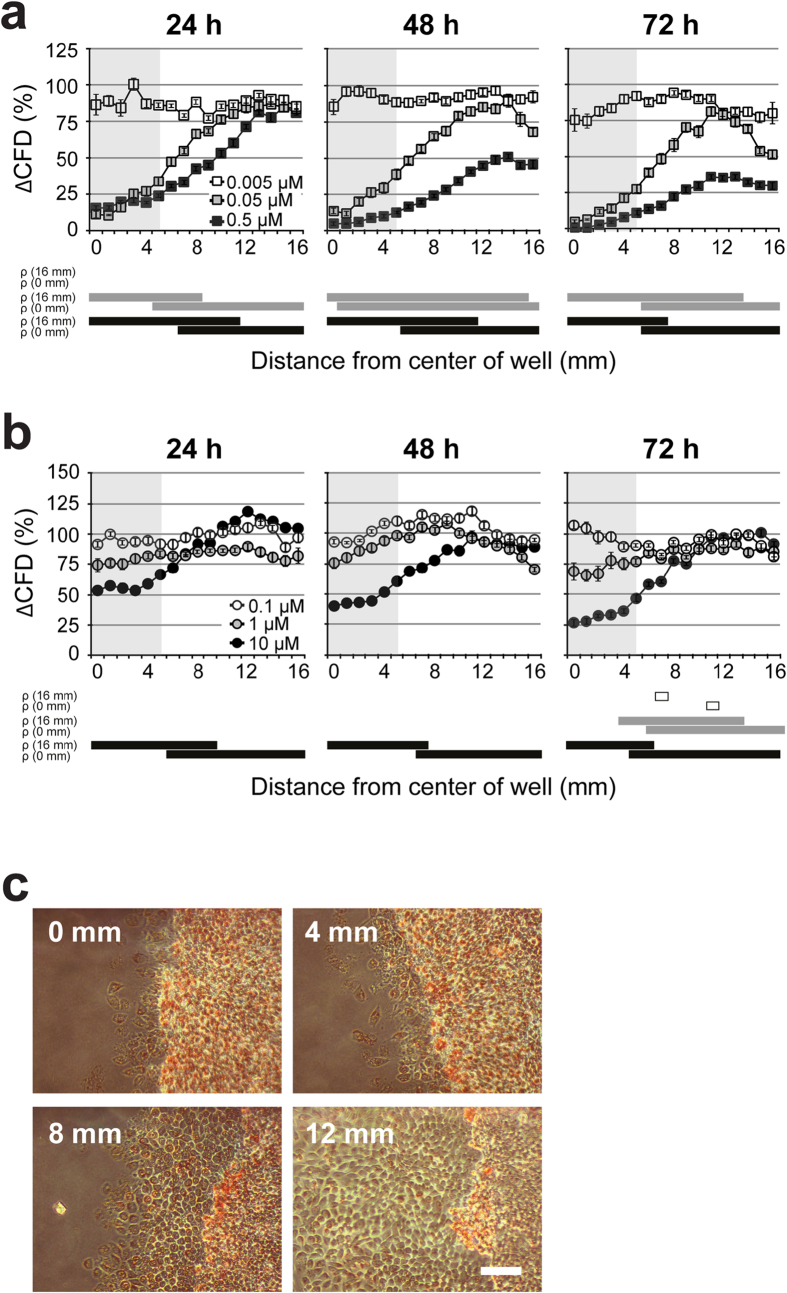
Cell migration indicates negative effects of bioactive compounds. Different concentrations of salinomycin (**a**) and cytochalasin D (**b**) were tested by the pellet method. For each time point, cell viability was assessed with NR staining. Migration distances were normalized to the corresponding control values. n = 3, data represents mean ± S.E.M. over the mean experimental data are indicated with error bars. The grey area in (**a**,**b**) indicates the locations covered by the agar pellet. The bars below the graph indicate *P* < 0.05 compared with the migration distance at 16 mm (ρ (16 mm)) or at 0 mm (ρ (0 mm)). Significant effects for 0.005, 0.05 and 0.5 μM salinomycin and 0.1, 1 and 10 μM cytochalasin D are indicated with white, grey and black bars, respectively. (**c**) Pictures were taken of cells, treated with 0.05 μM salinomycin for 72 h at the indicated distance from the center of the well. Scale bar is 100 μm.

Salinomycin and cytochalasin D are anti-tumorigenic primarily by interfering with cell migration. At these concentrations no cytotoxicity has been reported^30^,^31^,^32^. While in both cases the lowest evaluated concentration of the substances did not show any effect on CFD, a time- and concentration-dependent inhibition of CFD was observed at higher concentrations (Fig. 5a,b). Importantly, cell viability was not affected at any time point. However, morphological changes of the cells, like swelling and rounding up, can be observed concomitantly with the location of decreased migration (Fig. 5c).

**Figure 6:**
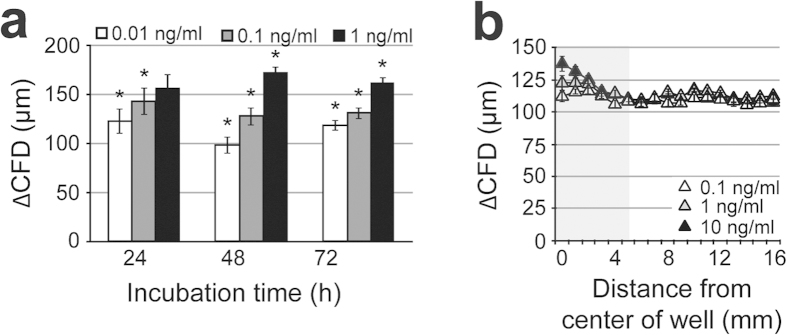
The agar diffusion scratch assay can also be used for bioactive migration stimulating drugs. Comparison of experiments in which bFGF was directly added to the agar overlay (**a**) and in the pellet placed on top of the agar overlay (**b**). bFGF applied in the agar overlay stimulates cell migration in a dose- and time-dependent manner. *Significant effect relative to the controls without test substance (*P *< 0.05). It also stimulated cell migration if tested according the agar diffusion scratch test using bFGF loaded pellets. In both cases migration was evaluated after 72 h of incubation. No effects on cell viability could be detected (data not shown). n = 3, data represents mean ± S.E.M. over the mean experimental data.

Although bFGF is known to promote both cell proliferation and migration^33^,^34^,^35^,^36^, it was shown for mouse fibroblasts to primarily enhance cell migration^37^. We tested its effect on CFD in two steps. First, we added bFGF at different concentrations directly within the agar used to overlay the cell culture. By that all cells were exposed to the same defined concentration of bFGF. Here an enhanced cell migration in a concentration- and time-dependent manner was observed (Fig. 6a). In a second step, the same compound concentrations were tested by the agar diffusion scratch method adding the compound solely in the pellet that was placed on top of the overlaid agar. Like in step 1, we observed a stimulation of cell migration defined by time point and concentration (Fig. 6b). However, the positive effect on cell migration only tends to be present for bFGF and is in this case not significant. In conclusion, our novel assay can identify compounds which are bioactive regarding stimulation or inhibition of cell migration but are not cytotoxic in the investigated concentration range.

## Discussion

Here we report the development of a novel method for drug and biomaterial evaluation combining in one test the advantages of the ISO10993-5 (biological evaluation of medical devices: Tests for *in vitro* cytotoxicity) and those of the wound healing assay. Drugs can be evaluated by this novel assay by loading the circular gel pellet with it and placing it on top of the agar overlay in analogy to a test biomaterial in the mentioned ISO guideline. Cell migration is a crucial phenomenon during all phases of life (e.g.^38^) including embryogenesis, homeostasis, tissue repair and immune surveillance. Failure of cell migration or inappropriate migration contributes to numerous severe pathological problems including vascular diseases, chronic inflammatory diseases, osteoporosis, cancer and mental retardation^13^. The migration of cells is a highly complex and subtly regulated process^39^,^40^ and it may be expected that changes in cell functionality will modify this. As a result cell migration has been subject of extensive research in the past and different assays have been developed to study this process, one of which being the scratch assay^21^.

In searching for the best agar concentration and volume data for the agar overlay of the cell cultures we could support those recommended by the ISO 10993-5 guidelines for the classical agar overlay test as being also optimal for the proposed newly developed test (3 ml of a 1% agar solution). While covering all positive aspects of the ISO10993-5 test, the agar diffusion scratch assay does not overcome all but still have some of its limitations: dilution of the bioactive substance by the agar, its eventual absorption by the agar matrix as well as the restrictions to either liquid or solid but not volatile test samples (reviewed in^2^). As for the agar overlay test, drawbacks of the scratch test are unfortunately also part of the new method. The restriction to adherent cells as well as the possible negative effect derived from damaged cells caused by scraping cannot completely be avoided. Furthermore, if a coating with extracellular matrix is performed, it may get damaged by the scraping (reviewed in^41^). In fact, surface coating has not been performed in this study and thus its resistance to scraping as well as its effect in the novel experimental set-up is assumed to be negligible but this still has to be confirmed.

Nevertheless, the combination of the agar overlay test and the scratch assay overcomes many limitations of the two underlying methods. These limitations can be summarized as follows:

- Due to the agar overlay the cells migrate compactly in the cell free area that was obtained according to the scratch assay. They form a clear migration front line enabling a clear definition of the migrated distance in contrast to the scratch assay since the border of the scratch stays clearly visible throughout the experiment. Hence it is possible to easily measure the migration distance by using basic tools provided with any basic microscopic software. This makes the scratch part of the novel assay technically much easier and cheap.
- The novel assay is highly reproducible and very easy to conduct. The use of a pipette tip may lead to unprecise generation of cell gaps; differences in the width of such small gaps cannot be completely avoided and may have an impact on the outcome of the experiment. In contrast, the use of a much wider scraper could rule out this limitation and increase the reproducibility within a series of experiments as well as between different laboratories adding additional strength to the proposed method.
- In contrast to the common scratch assay, the novel method can monitor cell migration for up to 72 h using the mouse fibroblast cell line L929. This allows examining the effect of a compound on cell migration for an extended time span compared to existing methods. By that also effects of compounds can be detected that act only with some delay. In addition, the novel method can be used like the scratch assay in both ways as a toxicity end-point as well as a kinetic assay.
- The integration of the agar overlay test renders it possible to not only investigate the effect on the ΔCFD of a single compound concentration but to assess it in relation to a decreasing gradient of test drug (or released biomaterial components) concentrations. A concentration-dependent effect on cell migration suggests pharmacokinetic properties of a given compound. In the same line, this novel method, unlike the scratch assay, allows evaluation of compounds in the field of chemotaxis.
- The novel assay with its use of small agar pellets allows an in depth evaluation of expensive and precious compounds by requiring only little quantities of the test substance.
- The evaluation of the novel method using pellets loaded with different kinds of drugs, i.e. cytotoxic substances and compounds which are bioactive at low and cytotoxic at high concentrations, revealed that with this novel set-up one can distinguish between pure cytotoxic chemicals (evaluated using CdSO_4_) from bioactive compounds (as evaluated here using salinomycin, cytochalasin D, and bFGF). In case of the cytotoxic compound CdSO_4_ effects on viability and migration were nearly overlapping. However, the evaluated bioactive compounds influenced ΔCFD (depending on the compound decreased or increased) in a dose- and time dependent way without affecting viability. By that evidence it is given that this novel method is able to sense and signal changes in a cell’s status in a more profound way than existing assays discriminating between cytotoxic and bioactive effects.

Thus, this novel assay represents a combination and further development of two very popular methods and consequently may open new avenues for parallel and simultaneous monitoring of toxic and bioactive compounds. This assay may not only bring many improvements to the *in vitro* biocompatibility testing but has in addition also great potential for other applications like tissue regeneration, chemotaxis, cancer, apoptosis and angiogenesis research.

## Methods

### Cell maintenance

Mouse fibroblast cell lines 3T3 NIH and L929 were maintained in Dulbecco’s modified Eagle medium: Nutrient mixture F-12 (DMEM/F-12) supplemented with 10% (v/v) foetal calf serum (FCS), 2 mM L-glutamine and 1% (v/v) penicillin and streptomycin (PenStrep) (all from Gibco). They were incubated in a humid chamber at 37 °C and 5% CO_2_. 5 × 10^5^ cells per well were seeded into 6-well plates (Sarstedt) 72 h prior testing in order to obtain a confluent monolayer.

### Scratching the cell layer and agar preparation

For the classical scratch asasay, a 200 μl pipette tip was used to obtain the cell free lane. A ruler was used as a guide to obtain a straight line. For the novel test we used PDMS/Sylgard 184 (Dow Corning, USA) to produce silicone scrapers with a bearing edge of 4 mm in order to generate wider cell free gaps. In order to remove the detached cells, the culture medium was discarded and the cells were washed twice with 3 ml of either DMEM/F-12 without supplements or 1x phosphate-buffered saline (PBS) (137 mM NaCl, 2.7 mM KCl, 10 mM Na_2_HPO_4_, 1.8 mM KH_2_PO_4_, 1 mM CaCl_2_·2H_2_O, 0.5 mM MgCl_2_·6H_2_O). After the last wash the remaining supernatant was removed with a micropipette tip and subsequently overlaid with 3 ml of 1% agar.

The agar was prepared as follows: 2% agar (Sigma-Aldrich) is prepared in water, autoclaved (20 min at 121 °C) and incubated in a water bath pre-warmed to 45 °C. After adapting the temperature to 45 °C, it is mixed with an equal volume of pre-warmed (45 °C) 2x DMEM/F-12 (Sigma-Aldrich) supplemented with 20% (v/v) FCS, 4 mM L-glutamine and 2% (v/v) PenStrep. The DMEM/F-12 is prepared from powder and sterilized by filtration (0.1 μm pore size, Millipore). Furthermore, it is favourable to use a medium lacking phenol red due to eventual colour interference with later neutral red staining.

Besides this we evaluated another methodology to obtain the cell-free lane. For this a silicon block was pressed on the cell culture dish before cell seeding with substratum contacting area 4 × 32 mm. However we found that the removal of the silicon block 24 h after seeding also resulted in the partial detachment of the contacting cell layer. Therefore the scratch technology was used further on for the agar diffusion scratch assay.

### Preparation of agar gel pellet containing the test substance

Bipartite Teflon moulds, fixed by rubber bands, were used to produce agar gel pellets of 1 cm diameter and 5 mm height (Supplementary Fig. 4). They were autoclaved (121 °C, 20 min) for sterilization purposes before moulding and transferred to −20 °C until usage. The agar pellets constitute of 2% agar and the test substance. CdSO_4_, salinomycin and cytochalasin D (all from Sigma-Aldrich) were diluted in H_2_O to the required concentration and added to appropriate aliquots of the agar solution (making up less than 3% of the final pellet volume). For the control samples we used agar pellets lacking any additional substance. For moulding, 400 μl of the agar-test sample mixture are pipetted into the moulds. Importantly, the moulds were pre-cooled prior moulding to −20 °C in order to accelerate the solidification process and as a result avoiding the spreading of the agar between the two moulding plate parts. The agar pellets are ready to use 5–10 min after moulding.

### Design of an evaluation grid label

In order to evaluate cell migration, we designed a transparent stick-on label for the bottom of the 6-well plates, which serves as a position grid to repeatedly monitor migration at a defined point (Supplementary Fig. 5). The grid lines run perpendicular to the scratch and are separated by 1 mm, every 4 mm being indicated by a red line. The numbers specify the distance from the centre of the grid, respectively the well. This labelling allows not only easy assessment of cell migration at a specific distance interval but also repeatedly at the same place of the gap at different time points of the experiment. The label was stuck to the plates directly after solidification of the agar. Alternatively, lines can be drawn by hand on the bottom of the well to evaluate cell migration at the same site throughout the experiment. However, regular and reproductive rasterizing of multiple plates will most likely not be possible manually.

### Culture treatment using agar overlay containing the test substance

In case the test substance was added directly into the agar overlay, the required amount of 1% agar mixture was transferred into a tube and the test substance was added. Thereby the volume of the test substance should be at maximum 10% of the agar volume in order to minimize changes of the agar concentration. In our test, lyophilized bFGF (Gibco) was reconstituted at 10 μg/ml in 1x PBS containing 0.1% BSA (Sigma-Aldrich) and further diluted to the required concentrations. After vortexing, the agar-test sample mixture was incubated at 45 °C until all bubbles disappeared. Finally, the cells were covered with the agar and the plates were left in the laminar flow until the agar was solidified and then incubated at cell culture conditions (37 °C and humidified air with 5% CO_2_). For the control, the cells were overlaid with agar that did not contain any additional substance. Cell migration and viability were evaluated after time points mentioned in the main text.

### Culture treatment using drug containing pellets

Centrally on each culture a pellet was carefully placed and gently pressed to remove any bubbles between the agar overlay and the pellet. Ultimately, the plates were incubated at cell culture conditions (37 °C and humidified air with 5% CO_2_). After time points mentioned in the main text cell migration and viability were evaluated.

### Evaluation of cell migration

Cell migration was evaluated after different incubation periods between 8 and 72 hours using an inverted microscope (CKX41, Olympus) connected to a camera (UC30, Olympus). Pictures were taken every millimeter on both sides of the scratch. Thus by placing the pellet in the middle of the well, four sets of results were obtained representing the 0–16 mm distance which can be taken together to calculate the mean value. In cases where the test substance was part of the agar overlay, pictures were taken every four millimeters on each side of the scratch and all migration values were taken into account for the mean value. The migration distance relative to the CFD at t = 0 h (ΔCFD) was determined with the help of the cellSens software (Olympus) by manually drawing a distance measuring line from the onset position of cell migration to the cell migration front. Two migration distances were measured per picture in order to have a representative value for each specific position along the scratch.

### Assessing cell viability by neutral red staining

Cell viability was measured using the Neutral Red (NR) uptake test. For this, NR (Sigma-Aldrich) was dissolved at 0.01% in 1x PBS and subsequently sterile-filtered. 3 ml of this solution were added to each well and incubated for 1 h at 37 °C. The staining solution was removed and the cells were incubated at 37 °C for 2–3 h to allow the stain to diffuse through the agar and to be taken up by the viable cells. Pictures were taken at each millimetre of the scratch of each well. Per area the number of stained (viable) and unstained (dead) cells was counted and percentage of viable cells determined. Once viability was determined, cultures could not be used anymore for further migration and life/dead analysis evaluation.

### Statistics

S-PLUS^®^ software was used to conduct the multiple comparison analysis of variance (ANOVA) using the Bonferroni *post-hoc* correction. In all instances in this study, an α level *P*-value of ≤0.05 was considered indicative of significance.

## Additional Information

**How to cite this article**: Pusnik, M. *et al.* The agar diffusion scratch assay - A novel method to assess the bioactive and cytotoxic potential of new materials and compounds. *Sci. Rep.* **6**, 20854; doi: 10.1038/srep20854 (2016).

## Supplementary Material

Supplementary Information
