# Supplementary material for: The agar diffusion scratch assay - A novel method to assess the bioactive and cytotoxic potential of new materials and compounds
Source: Sci Rep. 2016 Feb 10;6:20854. doi: 10.1038/srep20854 (PMC4748257; doi:10.1038/srep20854)
Supplement: Supplementary Information [file srep20854-s1.pdf]

# Supplementary Material

# The agar diffusion scratch assay - A novel method to assess the bioactive and cytotoxic potential of new materials and compounds

**Mascha Pusnik[1)], Minire Imeri[1)], Grégoire Deppierraz[1)], Arie Bruinink[2)*], and Manfred Zinn[1)*]**

[1)] Institute of Life Technologies, University of Applied Sciences and Arts Western Switzerland - HES-SO Valais-Wallis, Sion (CH)

[2)] Laboratory for Biointerfaces, Swiss Federal Laboratories for Materials Science and Technology - Empa, St. Gallen (CH)

*** Corresponding authors: arie.bruinink@empa.ch and manfred.zinn@hevs.ch**

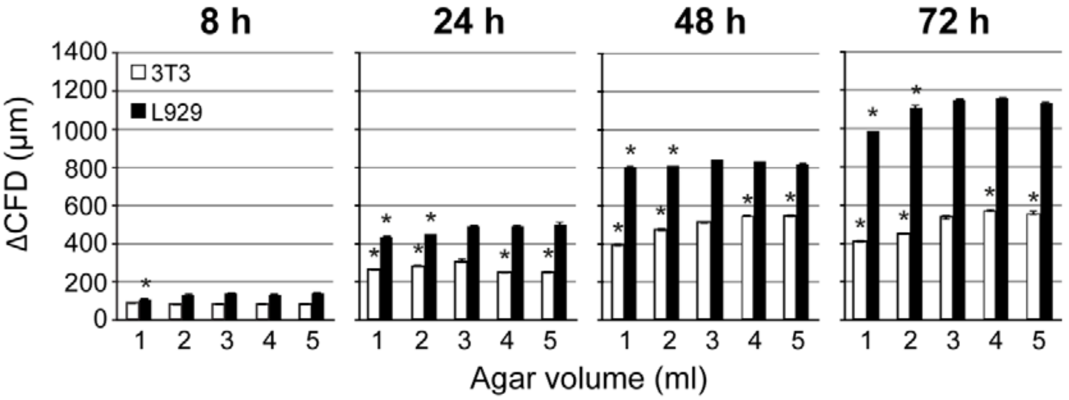


**Supplementary figure 1** Agar volume has a minor impact on cell migration distance. Mouse fibroblast 3T3 and L929 cell cultures (6-well plate) were covered with different volumes of 1% agar. ΔCFD's were assessed after 8, 24, 48 and 72 h of incubation. Measurements were done along the border lines of the scratch at intervals of 4 mm. *: Significant effects relative to value obtained for 3 ml agar of the corresponding cell line ($P < 0.05$, n = 3). Bars represent mean ± S.E.M. of mean values of 3 independent experiments.

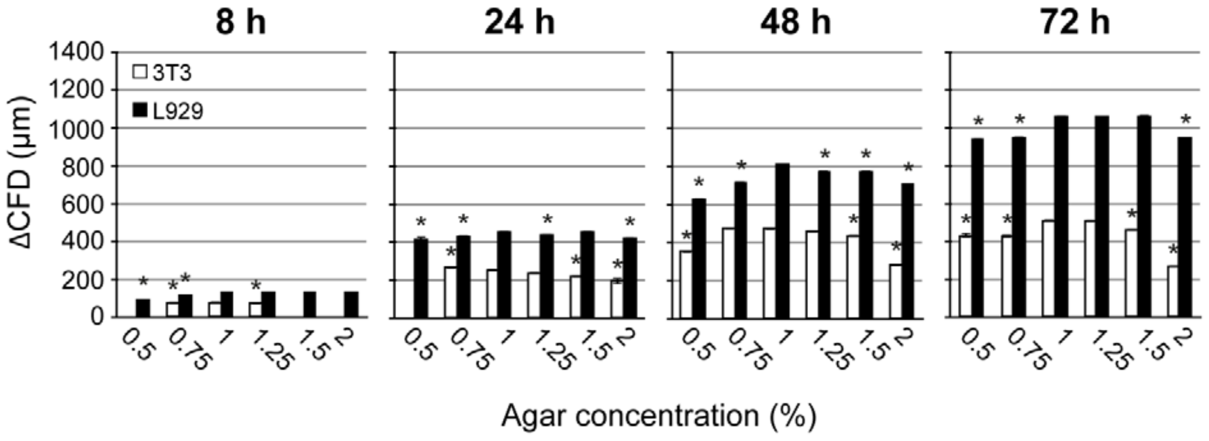


**Supplementary figure 2** Low and high concentrations of agar overlay resulted in reduced ΔCFD's. Mouse fibroblast 3T3 and L929 cell cultures were overlaid with 3 ml of 0.5-2% agar. ΔCFD's were assessed after 8, 24, 48 and 72 h of incubation. Measurements were done along the border lines of the scratch at intervals of 4 mm. *: Significant effects relative to value obtained for 1 % agar of the corresponding cell line ($P < 0.05$, n = 3). Bars represent mean ± S.E.M. of mean values of 3 independent experiments.

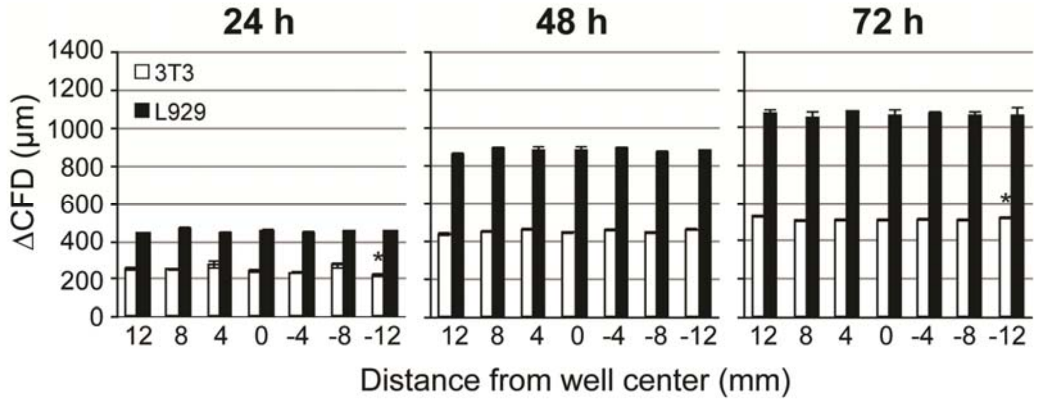


**Supplementary figure 3** Cell migration is uniform along the scratch. Mouse fibroblast 3T3 and L929 cell cultures were covered with 3 ml agar at 1% concentration. Measurements were done at the indicated distances along the scratch on both scratch sides starting from the dish centre. *: Significant effects relative to value obtained in the middle of the well (0 mm) of the corresponding cell line ($P < 0.05$, n = 3). Bars represent mean ± S.E.M. of mean values of 3 independent experiments.

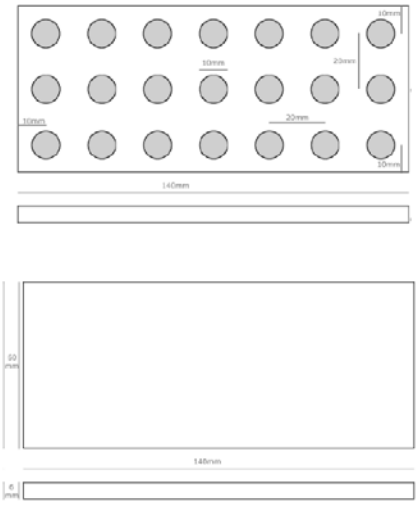


**Supplementary figure 4** Schematic representation of the Teflon moulds used for the production of the agar pellets. Upper and lower illustrations show the top and bottom part of the mould, respectively.

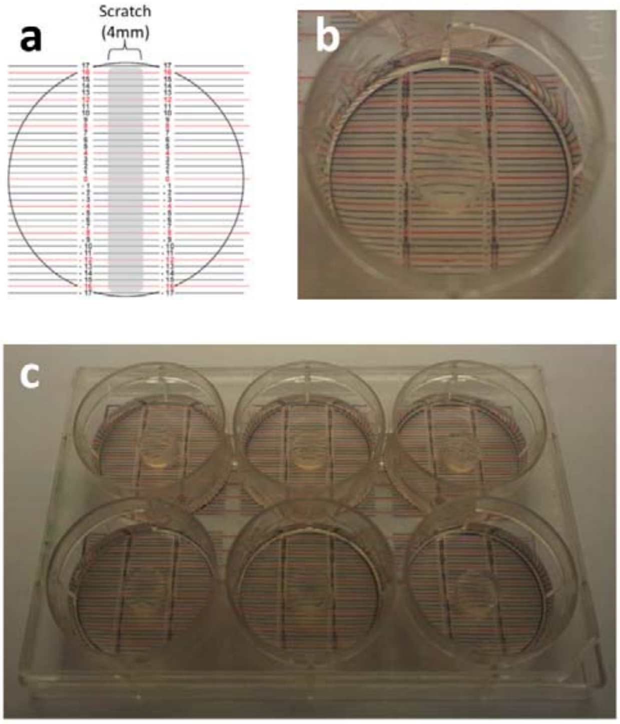


**Supplementary figure 5** Grid labels allow continuous tracking of migration at multiple time points. (a) Schematic representation of the sticker outline and the position of the scratch relative to the labelling. The numbers on both sides of the scratch indicated the distance from the well centre in millimeters. Every four millimeters the line was marked in red to facilitate orientation. (b) Picture of a well with distance marker lines of the sticker adhered to the bottom of the well. The well contained 3 ml of 1% agar. An agar gel pellet is placed on top the agar overlay in the well centre. (c) Picture of an entire 6-well plate with distance marker lines.
